# Chemical Constituents from the Aerial Parts of *Artemisia iwayomogi* and Their Anti-Neuroinflammatory Activities

**DOI:** 10.3390/plants11151954

**Published:** 2022-07-27

**Authors:** So-Ri Son, In Gyong Ju, Jinhee Kim, Keon-Tae Park, Myung Sook Oh, Dae Sik Jang

**Affiliations:** 1 Department of Biomedical and Pharmaceutical Sciences, Graduate School, Kyung Hee University, Seoul 02447, Korea; allosori@khu.ac.kr (S.-R.S.); jinhee1231@khu.ac.kr (J.K.); pgt940116@khu.ac.kr (K.-T.P.); 2 Department of Oriental Pharmaceutical Science, College of Pharmacy and Kyung Hee East-West Pharmaceutical Research Institute, Kyung Hee University, Seoul 02447, Korea; igju801@khu.ac.kr

**Keywords:** Compositae, *Artemisia iwayomogi*, sesquiterpene, neuroinflammation, nitric oxide

## Abstract

Neuroinflammation, predominantly mediated by microglial activation, is a key immunological response in the pathogenesis of neurodegenerative disorders. In our preliminary study, the aerial part of *Artemisia iwayomogi* inhibits LPS-induced microglial activation. The present study aims to identify chemical constituents with anti-neuroinflammatory properties in the aerial parts of *A*. *iwayomogi*. Two new guaianolide sesquiterpenes, iwayomogins A and B (**1** and **2**), along with thirteen known sesquiterpene lactones (**3**–**15**), one diterpene glycoside (**16**), and nine phenolic compounds (**17**–**25**) were isolated from the aerial parts of *A*. *iwayomogi* by repeated chromatography. The structures of the isolates were elucidated by their spectroscopic data. All isolates were evaluated for their inhibitory activities on nitric oxide (NO) production in LPS-induced BV-2 microglial cells. 2,3-Dehydro-1-*epi*-asperilin (**11**) exhibited the strongest inhibitory effect on NO production (IC_50_ value of 1.78 μM). In the molecular docking study, three compounds (**1**, **2**, and **11**) showed good binding affinities with iNOS. Additionally, compounds **1**, **2**, and **11** inhibit pro-inflammatory cytokines (TNF-α and IL-6) in dose-dependent manners. The present study demonstrates that the chemical constituents from *A*. *iwayomogi* inhibit NO production and pro-inflammatory cytokine release in BV-2 cells. However, further evaluation with biological experiments utilizing in vivo models is necessary.

## 1. Introduction

Neuroinflammation is an inflammatory response within the central nervous systems (CNS) which can be activated by a variety of neuronal insults, such as infection, trauma, and toxins [1]. Microglia, as the resident of macrophages in the CNS, induce a systematic neuroinflammation process that can cause neurodegenerative diseases including Alzheimer’s disease, Parkinson’s disease, multiple sclerosis, and prion disease [2]. When microglia are activated by various stimuli, including lipopolysaccharides (LPS), pro-inflammatory mediators are released, which can lead to neuronal death and neurogenesis inhibition [3]. For these reasons, to prevent neurodegenerative disorders, it is necessary to investigate the drugs that suppress the inflammatory mediators in microglia [4].

*Artemisia iwayomogi* Kitamura (synonym: *A. gmelinii* Weber ex Stechm.; Compositae; Dowijigi in Korean) is a perennial herb that has yellow-green leaves with a serrated shape [5]. The aerial parts of *A*. *iwayomogi* (Haninjin in Korean) have been used as a replacement for *Artemisia capillaris* Thunb. (Injinho in Korean), because of the similarities in name, appearance, and therapeutic efficacy, for a long time in Korea [6]. Both herbal medicines have been used to treat inflammatory mediated diseases in liver and skin, liver cirrhosis, jaundice, pruritus, and fever [7,8]. Recently, increasing scientific evidence revealed that *A*. *iwayomogi* has anti-inflammatory, anti-oxidative, anti-allergic, anti-obesity, and a CCl_4_-induced liver fibrosis inhibitory properties [9,10,11,12,13]. Notably, seco-guaianolide-type sesquiterpenes and coumarins isolated from *A*. *iwayomogi* inhibited inducible NOS (iNOS) expression in LPS-activated macrophages [14].

In our preliminary study, the EtOH extract from the aerial parts of *A*. *iwayomogi* significantly inhibited neuroinflammation induced by LPS in the murine microglial BV-2 cells of mice by suppressing pro-inflammatory mediators and NF-κB and MAPK pathways [15]. However, active compounds related to inflammation mediated CNS disorders have still not been detected in *A*. *iwayomogi*. Therefore, the present study aims to identify chemical constituents with anti-neuroinflammatory properties in the aerial parts of *A*. *iwayomogi*. In the present study, we conducted an investigation into novel bioactive compounds in the 90% EtOH extract of *A*. *iwayomogi* by repetitive chromatographic purification. To explore the inhibitory effects of the isolates on neuro-inflammation, we treated the compounds with BV-2 microglial cells and assessed their inhibitory activities on inflammatory mediators. We further investigated the interactions between iNOS with active compounds using molecular docking studies to determine whether they could be used in the treatment of NO-mediated inflammatory diseases.

## 2. Results and Discussion

### 2.1. Structure Elucidation of the Compounds Isolated from A. iwayomogi

**Figure 1:**
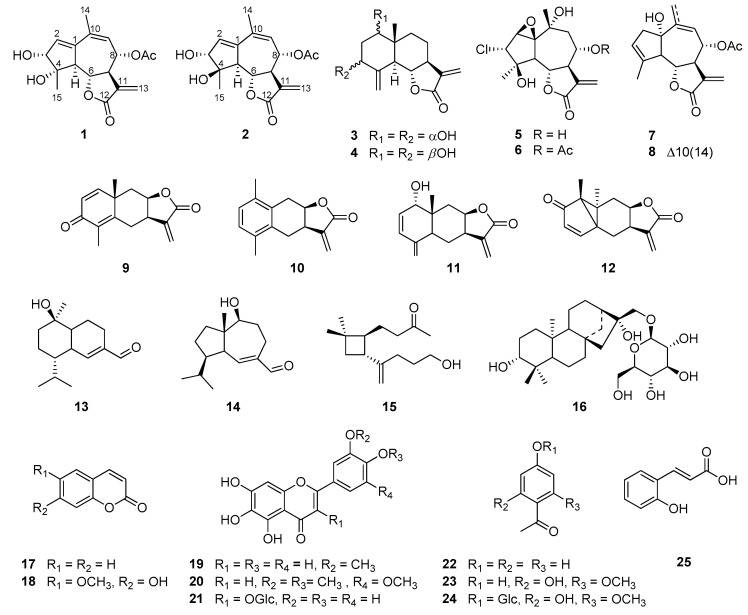
Chemical structures of compounds **1**–**25** isolated from the aerial parts of *A*. *iwayomogi*.

Repeated chromatography led to the isolation of two new guaianolide sesquiterpenes, iwayomogins A and B (**1** and **2**), along with ten sesquiterpene lactones (**3**–**12**), three sesquiterpenes (**13**–**15**), one diterpene glycoside (**16**), two coumarins (**17** and **18**), three flavonoids (**19**–**21**), and four phenolic compounds (**22**–**25**) from the aerial parts of *A*. *iwayomogi* (Figure 1).

**Figure 2:**
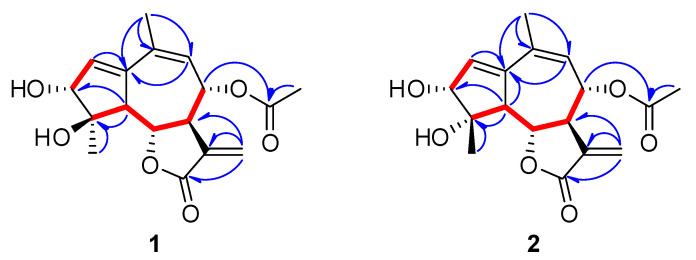
Key correlations observed in the COSY (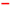) and HMBC (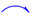) NMR spectra of **1** and **2**.

Compound **1** was isolated as a colorless solid, and its molecular formula was established as C_17_H_20_O_6_ by its pseudo-molecular ion peak HR-DART-MS (*m*/*z* 338.1601 [M+NH_4_]^+^; calcd for C_17_H_24_NO_6_, 338.1604) (Figure S1). The IR spectrum showed strong absorption bands at 1737 and 1660 cm^−1^, indicating the presence of *γ*-unsaturated lactone moiety (Figure S2). The ^1^H NMR spectrum of **1** (Table 1, Figure S3) also suggested the presence of an α-methylene-*γ*-lactone group through the diagnostic signals at *δ*_H_ 3.27 (1H, m, H-7), 4.35 (1H, dd, *J* = 11.5, 9.0 Hz, H-6), 5.76 (1H, d, *J* = 3.0 Hz, H-13a), and 6.22 (1H, d, *J* = 3.0 Hz, H-13b). Additionally, two olefinic protons [*δ*_H_ 5.52 (1H, br d, H-9), 6.21 (1H, t, *J* = 2.5 Hz, H-2)], two oxygenated methine protons [*δ*_H_ 3.95 (1H, d, *J* = 2.5 Hz, H-3), 5.50 (1H, dd, *J* = 11.5, 2.0 Hz, H-8)], a methine proton [*δ*_H_ 3.20 (1H, dd, *J* = 11.5, 2.5 Hz, H-5)], an acetyl proton [*δ*_H_ 2.17 (3H, s, 8-OCOCH_3_)], and two methyl proton signals [*δ*_H_ 1.32 (3H, s, H-15), 2.00 (3H, s, H-14)] were observed in the ^1^H NMR spectrum. The ^13^C NMR spectrum exhibited 17 carbon resonances, including 15 skeletal carbons and 2 characteristic carbons of an acetyl group [*δ*_c_ 21.2 (8-OCOCH_3_), 172.0 (8-OCOCH_3_)] (Table 1, Figure S4). With the assistance of the ^1^H-^13^C HSQC experiment (Figure S5), 15 skeletal carbon resonances including 4 *sp*^2^ quaternary carbons [*δ*_c_ 132.5 (C-10), 138.6 (C-11), 143.4 (C-1), 171.6 (C-12)], 2 olefinic carbons [*δ*_c_ 128.1 (C-9), 134.2 (C-2)], an α-methylene carbon [*δ*_c_ 123.5 (C-13)], 3 oxymethine carbons [*δ*_c_ 74.0 (C-8), 78.7 (C-6), 80.2 (C-4)], an oxygenated quaternary carbon [*δ*_c_ 80.4 (C-3)], 2 methine carbons [*δ*_c_ 50.0 (C-7), 56.3 (C-5)], and 2 methyl carbons [*δ*_c_ 21.8 (C-15), 25.4 (C-14)] were interpreted. The ^1^H-^1^H COSY spectrum exhibited correlations between H-2 and H-3 and continuous correlations from H-5 to H-9 (Figure 2 and Figure S6). Combined with the above results and the ^1^H-^13^C HMBC signals (H-2/C-1; H-5/C-1, C-3, C-4; H-9/C-1, C-10; H-14/C-1, C-10, C-9; H-15/C-4, C-5), it was suggested that **1** is a guaianolide-type sesquiterpene with a 5/7/5 tricyclic system (Figure 2 and Figure S7). Moreover, the long-range correlations between H-8 and the carbonyl carbon of the acetyl group indicated that the *O*-acetyl group was located at C-8. Thus, the plain structure of compound **1** was almost identical to artemdubolide F except for the presence of an acetoxy group at C-8 [16].

**Figure 3:**
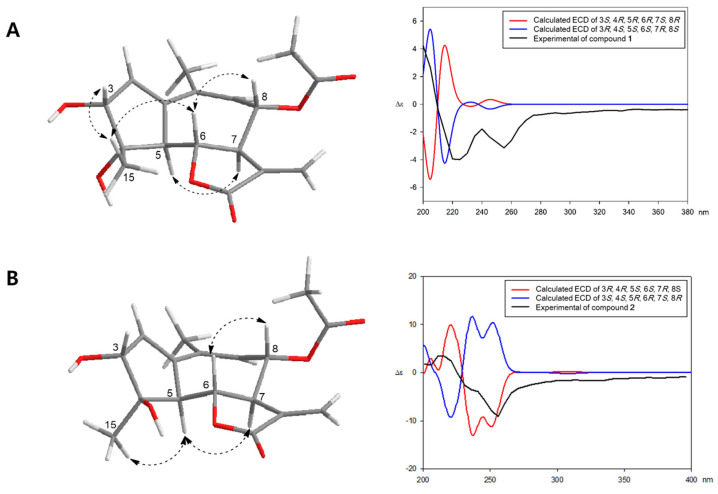
Key NOESY correlations and comparison of experimental and calculated ECD spectra of compounds **1** (**A**) and **2** (**B**).

The relative configuration of compound **1** was determined by the coupling constants and NOESY experiment (Figure 3 and Figure S8). Through the large coupling constants (*J*_5,6_ = 11.5 Hz, *J*_6,7_ = 9.0 Hz, and *J*_7,8_ = 11.5 Hz), we confirmed that H-5, H-6, H-7, and H-8 had *trans*-diaxial configurations. Starting from *β*-oriented H-6, the NOESY correlations of H-6/H-8, H-6/H-15, and H-3/H-15 suggested that H-3, H-6, H-8, and H-15 are located on the *β*-side of the ring system. Additionally, the cross-peak between H-5 and H-7 was observed in the NOESY spectrum of **1**, indicating that they are located on the *α*-side of the ring system (Figure 3). To determine the absolute configuration, we compared the experimental ECD spectrum of **1** with the calculated ECD data of two enantiomeric models 3*R*/4*S*/5*S*/6*S*/7*R*/8*S* and 3*S*/4*R*/5*R*/6*R*/7*S*/8*R* using the TDDFT method. The calculated ECD data of the 3*R*/4*S*/5*S*/6*S*/7*R*/8*S* configuration was in good agreement with the experimental data of **1** (Figure 3). Therefore, the structure of **1** was elucidated as (3*R*,4*S*,5*S*,6*S*,7*R*,8*S*)-8*α*-acetoxy-3*α*,4*α*-dihydroxy-guaia-1,9,11(13)-trien-12,6*α*-olide, and named iwayomogin A, as shown in Figure 1.

Compound **2** exhibited the same pseudo-molecular ion peak as compound **1** in the HR-DART-MS spectrum (*m*/*z* 338.1601 [M+NH_4_]^+^; calcd for C_17_H_24_NO_6_, 338.1604) (Figure S9). The IR spectrum of **2** also revealed the presence of a γ-unsaturated lactone group (1741 and 1661 cm^−1^) (Figure S10). The ^1^H and ^13^C NMR spectra of **2** showed strong similarities with those of **1** (Table 1, Figures S11 and S12). However, one methyl proton signal [*δ*_H_ 1.54 (3H, s, H-15)] and two oxygenated methine signals [*δ*_H_ 4.09 (1H, d, *J* = 3.0 Hz, H-3) and 4.41 (1H, d, *J* = 11.0, 9.0, H-6)] of **2** were moved to the downfield region, whereas one methine signal [*δ*_H_ 3.05 (1H, dd, *J* = 11.0, 2.5, H-5)] was shifted to the upfield area. Analysis of the ^13^C NMR and HSQC experimental data (Table 1, Figure 2, Figures S12 and S13) determined that the C-5 methine signal (*δ*_c_ 59.6) of **2** was deshielded, whereas the H-5 methine signal was shielded compared with those of **1**. The carbon signals at C-3, C-4, C-6, and C-15 of **2** were shifted to the downfield region. The key NOESY correlations of H-5/H-15, H-5/H-17, and H-6/H-8 indicated that compound **2** should be the 4-epimer of **1** (Figure 3 and Figure S16). The absolute configuration of **2** was confirmed by comparing the calculated ECD spectra and the experimental spectrum (Figure 3). Thus, the chemical structure of **2** was determined as (3*R*,4*R*,5S,6*S*,7*R*,8*S*)-8*α*-acetoxy-3*α*,4*ß*-dihydroxy-guaia-1,9,11(13)-trien-12,6*α*-olide, and named iwayomogin B.

The known compounds were identified as ludovicin B (**3**) [17], ridentin B (**4**) [18], bibsanin (**5**) [19], bibsanin monoacetate (**6**) [20], lupicolin A acetate (**7**) [21], lupicolin B acetate (**8**) [21], yomogin (**9**) [22], 10-desmethyl-1-methyl-5,6-dihydroeudesma-1,3,5(10)-triene-12,8*β*-olide (**10**) [23], 2,3-dehydro-1-*epi*-asperilin (**11**) [23], lumiyomogin (**12**) [23], 10*α*-hydroxy-cadin-4-en-15-al (**13**) [24], 10*β*-hydroxyisodauc-6-en-14-al (**14**) [25], 5-hydroxy-5,6-secocaryophyllen-6-on (**15**) [26], iwayoside A (**16**) [27], coumarin (**17**) [28], scopoletin (**18**) [28], jaceosidin (**19**) [29], arteanoflavone (**20**) [30], patuletin 3-*O*-*β*-d-glucoside (**21**) [31], *p*-acetophenol (**22**) [32], 2,4-dihydroxy-6-methoxy acetophenone (**23**) [33], annphenone (**24**) [33], and *O*-coumaric acid (**25**) [34] based on previously reported data (Figure 1). Among the isolates, ludovicin B (**3**), ridentin B (**4**), bibsanin (**5**), bibsanin monoacetate (**6**), yomogin (**9**), 10-desmethyl-1-methyl-5,6-dihydroeudesma-1,3,5(10)-triene-12,8*β*-olide (**10**), 2,3-dehydro-1-*epi*-asperilin (**11**), lumiyomogin (**12**), 10*α*-hydroxy-cadin-4-en-15-al (**13**), 10*β*-hydroxyisodauc-6-en-14-al (**14**), and 5-hydroxy-5,6-secocaryophyllen-6-on (**15**) were isolated from *A*. *iwayomogi* for the first time, to the best of our knowledge.

### 2.2. Inhibitory Effects of the Isolates on NO Production

Nitric oxide (NO) is one of the crucial signaling molecules that mediates the inflammatory processes [35]. In order to determine which isolates from *A*. *iwayomogi* exert regulatory activities on NO production, all isolates were treated with LPS on BV-2 microglial cells and the concentration of NO in the supernatant of the treated cells was measured. 10-Desmethyl-1-methyl-5,6-dihydroeudesma-1,3,5(10)-triene-12,8*β*-olide (**10**) and lumiyomogin (**12**) were excluded from the comparison because of their considerable cytotoxicity at 10 μM concentration, although none of the other compounds showed significant cytotoxicity.

As shown in Table 2, ludovicin B (**3**), bibsanin (**5**), bibsanin monoacetate (**6**), lupicolin A acetate (**7**), yomogin (**9**), and 2,3-dehydro-1-*epi*-asperilin (**11**) showed strong NO inhibitory activities, with IC_50_ values less than 10 μM. Among them, 2,3-dehydro-1-*epi*-asperilin (**11**) was the most potent NO inhibitor, with an observed IC_50_ value of 1.78 μM. The new guaianolide-type sesquiterpenes, iwayomogins A and B (**1** and **2**), also showed moderate NO inhibitory activity, with IC_50_ values of 10.59 and 12.85 μM, respectively. Our findings imply that these sesquiterpene lactones are the active compounds responsible for the anti-neuroinflammatory properties of *A*. *iwayomogi*.

### 2.3. Molecular Docking Studies of the Active Compounds

**Figure 4:**
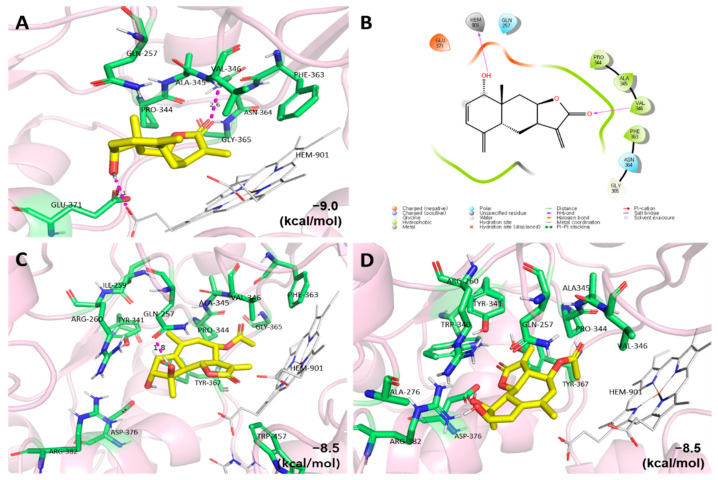
The 3D and 2D diagrams of molecular docking simulations were obtained at the lowest energy conformation (RMSD < 1.0), highlighting potential hydrogen contact for compounds **11** (**A**), **1** (**C**), and **2** (**D**), respectively. Colored by atom: carbon is yellow/green; nitrogen is blue; oxygen is red. For clarity, only the interacting residues are labeled. Hydrogen bonding interactions are shown by dashes. Further analysis of the interactions between iNOS and compound **11** is shown (**B**). Visual illustrations of the interactions were created by PyMOL and Maestro.

NO is synthesized by three types of nitric oxide synthase with L-arginine and cofactors, and iNOS plays an important role in the NO production process [36]. Therefore, selective iNOS inhibitory agents have been studied by several researchers [37]. To further investigate the effects of the compounds reducing NO concentration on the inhibition of iNOS protein (PDB code: 3E6T), the most active compound (**11**) and the new compounds (**1** and **2**) were selected for an in silico molecular docking study (Figure 4). Compounds **11**, **1**, and **2** showed strong binding affinities (−9.0, −8.0, and −8.0 kcal/mol, respectively) with iNOS. At the lowest energy binding mode, compound **11** interacted with both ALA-345 and HEM-901, which are involved in hydrogen bonding. In addition, compound **1** interacted with GLN-257 via hydrogen bonding, although the binding affinities were the same in the lowest docking conformation for compounds **1** and **2**. The data also suggests that the active compounds may reduce NO production in BV-2 microglial cells by inhibiting iNOS activity.

### 2.4. Inhibitory Effects on Pro-Inflammatory Cytokines

**Figure 5:**
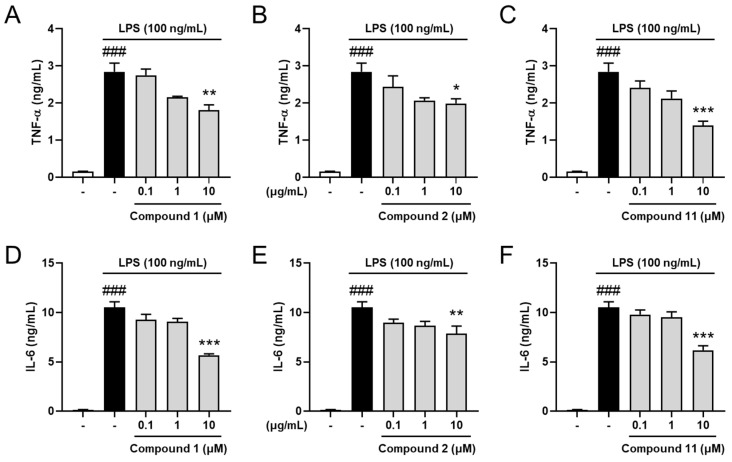
Effects of compounds **1**, **2**, and **11** on pro-inflammatory cytokine release in LPS-stimulated BV-2 cells. The cells were pre-treated with compounds **1** (**A**,**D**), **2** (**B**,**E**), or **11** (**C**,**F**) for 1 h before LPS treatment. At 23 h after LPS stimulation, the supernatants were collected, and TNF-α (**A**–**C**) and IL-6 (**D**–**F**) were measured using ELISA kits. Control group, vehicle-only-treated group (white bar); LPS-treated group (black bar); LPS with compounds-treated groups (gray bars). Values are indicated as the mean ± SEM. Data were analyzed by one-way ANOVA, followed by Tukey’s post-hoc test. ### *p* < 0.001 compared with the control group; * *p* < 0.05, ** *p* < 0.01, and *** *p* < 0.001 compared with the LPS-only-treated group.

TNF-α and IL-6 are typical pro-inflammatory cytokines, which contribute to inflammatory propagation and aggravation [38,39]. To investigate the molecular mechanism of the most potent compound, 2,3-dehydro-1-*epi*-asperilin (**11**), and the new compounds—iwayomogins A and B (**1** and **2**)—on neuroinflammatory response, the concentrations of the pro-inflammatory cytokines in the supernatant of the treated cells were measured. The results showed that TNF-α and IL-6 levels were markedly elevated in the LPS-only-treated group compared with the control group. However, compounds **1**, **2**, and **11** reduced cytokine concentrations in dose-dependent manners (Figure 5). These results indicate that compounds **1**, **2**, and **11** are effective to control neuroinflammation via simultaneous regulation of pro-inflammatory mediators. These effects are thought to be related to interactions with iNOS and the consequent regulation of NO production, resulting in the cessation of the overall inflammatory process, including the release of cytotoxic cytokines. Considering that neuroinflammation is one of the contributors in various psychological disorders and neurodegenerative diseases, compounds **1**, **2**, and **11** are anticipated to be potential candidates for treatment of neurological disorders due to their anti-neuroinflammatory effects.

## 3. Materials and Methods

### 3.1. General Experimental Procedure

Diaion HP-20 resin (Mitsubishi Chemical Industries, Ltd., Tokyo, Japan), Sephadex LH-20 gel (GE Healthcare, Stockholm, Sweden), silica gel (230–400 mesh, Merck, Kenilworth, MA, USA), and LiChroprep RP-18 gel (40–63 mm, Merck) were used for open CC. Fractions were monitored by TLC analyses on silica gel 60 F_254_ (Merck) and RP-18 F_254S_ (Merck) plates. MPLC (Teledyne Isco, Lincoln, NE, USA) was used for further fractionation with pre-packed Redi Sep-Silica (12 g, 24 g, 40 g, Teledyne Isco) and Redi Sep-C18 cartridges (26 g, 43 g, 80 g, Teledyne Isco). Preparative HPLC was performed using a HPLC purification system (1525 pump and PDA 1996 detector, Waters Corp., Milford, MA, USA) with a Gemini NX-C18 110A (250 × 21.2 mm i.d., 5 μm, Phenomenex, Torrance, CA, USA) or YMC-pack ODS-A columns (250 × 20.0 mm i.d., 5 μm, YMC Co., Ltd., Kyoto, Japan). UV and FT-IR spectra were recorded using OPTIZEN UV–Vis and Agilent Cary 630 FT-IR (Agilent Technologies, Santa Clara, CA, USA) spectrophotometers, respectively. Optical rotations were measured using a JASCO P-2000 polarimeter; NMR spectra were acquired by JEOL (JEOL, Tokyo, Japan) at 500 MHz; and HR-DART-MS spectra were obtained by the DART ion source (Ionsense, Tokyo, Japan) coupled to an AccuTOF-TLC (JEOL). Finally, the circular dichroism spectra were measured by a Chirascan-plus spectrometer (Applied Photophysics Ltd., Leatherhead, UK).

### 3.2. Plant Material

The aerial parts of *Artemisia iwayomogi* Kitamura (Compositae) were purchased from Kwangmyoungdang Pharmaceutical Co., Ltd. (Ulsan-si, Korea) in January 2019. The origin of the herbal material was identified by Professor Myeong Sook Oh, and a voucher specimen (ARIW-2019) was deposited in the Laboratory of Natural Product Medicine, College of Pharmacy, Kyung Hee University (Seoul, Korea).

### 3.3. Extraction and Isolation

The dried plant materials (3.0 kg) were extracted twice with 90% EtOH (30 L) over the course of 48 h at room temperature, then the solvent was removed in vacuo at 45 °C to give a 90% EtOH extract (300 g). The extract was then suspended in distilled water (0.6 L) and partitioned three times with EtOAc (0.6 L). The EtOAc-soluble fraction (130 g) was subjected to Diaion HP-20 CC (11.3 × 55.6 cm) using an acetone-water gradient system (from 30:70 to 100:0, *v*/*v*) to obtain 19 fractions (F1–F19). Compound **18** (1.59 g) was purified by recrystallization from F3 in cold acetone. After recrystallization, the supernatant of F3 (18.1 g) was subjected to Sephadex LH-20 CC (5.5 × 61.0 cm; acetone-H_2_O = 45:55, *v*/*v*) to produce 13 subfractions (F3-1–F3-13). Subfraction F3-3 was fractionated further by silica gel CC (230–400 mesh; 5.3 × 34.0 cm; *n*-hexane-EtOAc-MeOH = 60:35:5 to 0:95:5, *v*/*v*/*v*) to purify compounds **1** (2.2 mg), **2** (5.6 mg), **3** (6.2 mg), **5** (1 mg), **16** (10 mg), and **24** (290.4 mg). F3-6 was fractionated by silica gel CC (230–400 mesh; 5.3 × 34.0 cm; *n*-hexane-EtOAc = 75:25 to 0:100, *v*/*v*) to give compounds **21** (249.8 mg) and **22** (243.8 mg). F6 (3.5 g) was separated by Sephadex LH-20 CC (3.0 × 42.0 cm, MeOH-H_2_O = 55:45, *v*/*v*) to isolate compounds **4** (4.0 mg), **6** (10.8 mg), **17** (166.2 mg), and **25** (213.8 mg). Compound **9** (0.98 g) was purified by recrystallization from F7 in MeOH and H_2_O (1:1). The supernatant of F7 (7.78 g) was then fractionated further by Sephadex LH-20 CC (5.5 × 61.0 cm) with MeOH-H_2_O (8:2, *v*/*v*) to yield nine subfractions (F7-1–7-9). Compounds **7** (5.6 mg), **8** (6.0 mg), **11** (5.0 mg), and **12** (5.0 mg) were obtained from F7-3 by silica gel CC (230–400 mesh; 5.3 × 34.0 cm; *n*-hexane-EtOAc = 70:30 to 0:100, *v*/*v*) followed by preparative HPLC on a Gemini NX-C18 110A column (MeOH-H_2_O = 35:65 to 70:30, *v*/*v*). Fraction 8 (4.24 g) was separated into 13 subfractions (F8-1–8-13) by Sephadex LH-20 CC (3.0 × 70.0 cm, acetone-H_2_O = 70:30, *v*/*v*). Compound **19** (25.2 mg) was purified by recrystallization from F8-13 in MeOH. F8-7 was separated using silica gel CC (230–400 mesh; 3.0 × 25.0 cm; *n*-hexane-EtOAc-MeOH = 80:15:5 to 70:25:5, *v*/*v*) to give compound **23** (90.2 mg). F11 (1.40 g) was subjected to Sephadex LH-20 CC (5.5 × 65.0 cm; MeOH-H_2_O = 80:20, *v*/*v*) to obtain compounds **13** (3.5 mg), **14** (4.8 mg), and **15** (3.1 mg). F13 (2.09 g) was separated into ten subfractions (F13-1–13-10) by Sephadex LH-20 CC (3.0 × 63.0 cm, acetone-H_2_O = 70:30, *v*/*v*). Compounds **10** (25.3 mg) and **20** (6.2 mg) were isolated from F13-6 by reversed-phase MPLC with a Redi Sep-C18 cartridge (60 g, MeOH-H_2_O, from 65:35 to 85:15, *v*/*v*).

#### 3.3.1. Iwayomogin A (**1**)

Colorless solid; HR-DART-MS (positive mode) *m*/*z* = 338.1601 [M+NH_4_]^+^ (calcd for C_17_H_24_NO_6_, 338.1604) ${\left[\alpha \right]}_{D}^{20}$: +173 (c 0.01, MeOH); UV (MeOH) λ_max_ nm (log ε): 241 (3.85); IR (ATR) ν_max_ 3313, 2929, 1741, 1661 cm^−1^; ^1^H and ^13^C NMR data (see Table 1).

#### 3.3.2. Iwayomogin B (**2**)

Colorless solid; HR-DART-MS (positive mode) *m*/*z* = 338.1601 [M+NH_4_]^+^ (calcd for C_17_H_24_NO_6_, 338.1604) ${\left[\alpha \right]}_{D}^{20}$: +41 (c 0.01, MeOH); UV (MeOH) λ_max_ nm (log ε): 242 (3.86); IR (ATR) ν_max_ 3391, 2928, 1737 cm^−1^; ^1^H and ^13^C NMR data (see Table 1).

### 3.4. Computational Methods for the ECD Spectrum

The 3D models of active compounds were built using Chem3D, and the random conformational analysis was performed with the Merk molecular force field (MMFF) implemented by the Spartan’14 software program (Wavefunction, Inc., Irvine, CA, USA; 2014). Geometrical optimization of the selected lowest energy conformers was performed at the B3YLP/6-31+g(d, p) level using Gaussian 09 (Revision E.01; Gaussian, Inc., Wallingford, CT, USA; 2009). The electronic circular dichroism (ECD) calculations of the optimized conformers were calculated using the time-dependent density functional theory (TDDFT) method at the CAM-B3LYP/SVP level with the conductor-like polarizable continuum solvent model (CPCM, methanol). The final ECD spectra were generated by Boltzmann weighting each conformer.

### 3.5. Cell Culture and Treatment

BV-2 microglial cells were maintained in Dulbecco’s Modified Eagle’s medium (Hyclone Laboratories, Inc., Logan, UT, USA), supplemented with 10% fetal bovine serum (Hyclone Laboratories) and 1% penicillin-streptomycin (Hyclone Laboratories), and incubated at 37 °C in a humidified atmosphere containing 5% CO_2_. All experiments were carried out 24 h after seeding in 96-well or 12-well plates at a density of 3.0 × 10^5^ cells/mL. The following day, the cells were pre-treated with various concentrations of the compounds in serum-free media for 1 h, before being stimulated with 100 ng/mL of LPS (Sigma-Aldrich, St. Louis, MO, USA) for an additional 23 h. An equal volume of vehicle was given to the control and each of the toxin groups.

### 3.6. Measurement of Pro-Inflammatory Mediators

NO concentration was determined using the method described in the previous study [15]. The supernatant of the cells seeded in a 96-well plate was harvested and mixed with an equal volume of Griess reagent (1% sulfanilamide, 0.1% naphthylethylenediamine dihydrochloride, 2% phosphoric acid). After 10 min, the absorbance at 540 nm was measured using a microplate reader (Versamax™, Molecular Devices, LLC., San Jose, CA, USA). Sodium nitrite was used as a standard to calculate the NO concentration. TNF-α (BD Biosciences, Franklin Lakes, NJ, USA) and IL-6 (R&D Systems, Minneapolis, MN, USA) protein concentrations in the supernatant of cells seeded in the 12-well plates were assessed using enzyme-linked immunosorbent assay (ELISA) kits following the manufacturer’s protocols.

### 3.7. Molecular Docking

The crystal structures of iNOS were obtained from the RCSB PDB database (PDB code: 3E6T, resolution: 2.5 Å). The protein was prepared by removing all water molecules and adding polar hydrogen atoms using AutoDock 4.2 software (The Scripps Research Institute, La Jolla, CA, USA). The grid box size was 30 Ǻ ×30 Ǻ ×30 Ǻ with 0.175 Ǻ. The absolute configurations of compounds **1** and **2** were confirmed by NOESY and ECD calculation data, and the 3D structures of the ligands were minimized using Chem3D Pro 14.0. After the preparation of the protein and ligands, molecular docking calculations were performed using AutoDock Vina software with AutoDock Tools 1.5.6. (The Scripps Research Institute, La Jolla, CA, USA) and using the hybrid Lamarckian Genetic Algorithm (LGA). The 2D and 3D diagrams of the protein–ligand complexes and the 2D diagrams of protein–ligand interactions were generated with Maestro 12.9 software (Schrödinger, LLC., New York, NY, USA).

### 3.8. Statistical Analysis

Statistical parameters were calculated using GraphPad Prism 8.0 software (GraphPad Software, San Diego, CA, USA). Values were expressed as the mean ± standard error of the mean (SEM) and analyzed using a one-way analysis of variance (ANOVA) followed by Tukey’s post-hoc test. Differences with *p*-values of less than 0.05 were considered statistically significant.

## 4. Conclusions

The aerial parts of *A. iwayomogi* have been widely used in traditional Korean medicine to treat various inflammation-mediated diseases. Our findings revealed that the chemical constituents from *A. iwayomogi* can inhibit neuroinflammation in LPS-induced BV-2 cells by suppressing the NO production and the release of pro-inflammatory cytokines. Considering that neuroinflammation contributes to various psychological disorders and neurodegenerative diseases, these isolates could be used in the treatment of neurological disorders because of their anti-neuroinflammatory properties. Therefore, further research is necessary to confirm whether their anti-inflammatory effects would be effective against neurological disorders in in vivo models.

## Figures and Tables

**Table 1:** ^1^H- and ^13^C-NMR spectroscopic data of compounds **1** and **2**.

Position *^a^*	1 *^c^*		2 *^c^*	
	*δ*_H_ *^b^*	*δ*_C_	*δ*_H_ *^b^*	*δ*_C_
1		143.4		143.5
2	6.21 t (2.5)	134.2	6.19 t (2.5)	134.9
3	3.95 d (3.0)	80.4	4.09 d (3.0)	83.4
4		80.2		82.2
5	3.20 dd (11.5, 2.5)	56.3	3.05 dd (11.0, 2.5)	59.6
6	4.35 dd (11.5, 9.0)	78.7	4.41 (11.0, 9.5)	79.2
7	3.27 m	50.0	3.34 m	49.5
8	5.50 dd (11.5, 2.0)	74.0	5.47 dd (11.0, 2.0)	74.0
9	5.52 br d	128.1	5.50 d (2.0)	129.8
10		132.5		132.3
11		138.6		138.6
12		171.6		171.7
13	5.76 d (3.0) 6.22 d (3.0)	123.5	5.78 d (3.0) 6.22 d (3.0)	123.6
14	2.00 s	25.4	2.03 s	26.0
15	1.32 s	21.8	1.54 s	23.1
8-OCOCH_3_	2.17 s	21.2	2.17 s	21.2
8-OCOCH_3_		172.0		172.0

*^a^* All assignments were based on ^1^H-^1^H COSY, ^1^H-HSQC, and ^1^H-^13^C HMBC. *^b^ δ*_H_ Multi (*J* in Hz). *^c^*Measured in methanol-*d*_4_.

**Table 2:** IC_50_ values of compounds isolated from the aerial parts of *A*. *iwayomogi* inhibiting NO production in BV-2 cells.

Compound	IC_50_ (μM) *^a^*	Compound	IC_50_ (μM) *^a^*
**1**	10.59	**15**	>30
**2**	12.85	**16**	>30
**3**	3.83	**17**	>30
**4**	13.59	**18**	19.91
**5**	8.59	**19**	19.25
**6**	8.29	**20**	8.08
**7**	7.38	**21**	>30
**8**	14.50	**22**	>30
**9**	5.24	**23**	22.52
**11**	1.78	**24**	>30
**13**	>30	**25**	>30
**14**	>30	Quercetin *^b^*	8.14

*^a^* IC_50_ values for the inhibition of NO production were determined in LPS-treated BV-2 cells in comparison to cells treated with the vehicle. *^b^* Quercetin was used as a positive control. All values were calculated as the mean of results by performing experiments in triplicate.

## Data Availability

Not applicable.

## Supplementary Materials

The following supporting information can be downloaded at: https://www.mdpi.com/article/10.3390/plants11151954/s1, Figure S1. HR-DART-MS spectrum of compound **1**; Figure S2. IR spectrum of compound **1**; Figure S3. ^1^H NMR spectrum of compound **1** (500 MHz, methanol-*d*_4_); Figure S4. ^13^C NMR spectrum of compound **1** (125 MHz, methanol-*d*_4_); Figure S5. ^1^H ^13^C HSQC spectrum of compound **1**; Figure S6. ^1^H ^1^H COSY spectrum of compound **1**; Figure S7. ^1^H ^13^C HMBC spectrum of compound **1**; Figure S8. ^1^H ^1^H NOESY spectrum of compound **1**; Figure S9. HR-DART-MS spectrum of compound **2**; Figure S10. IR spectrum of compound **2**; Figure S11. ^1^H NMR spectrum of compound **2** (500 MHz, methanol-*d*_4_); Figure S12. ^13^C NMR spectrum of compound **2** (125 MHz, methanol-*d*_4_); Figure S13. ^1^H ^13^C HSQC spectrum of compound **2**; Figure S14. ^1^H ^1^H COSY spectrum of compound **2**; Figure S15. ^1^H ^13^C HMBC spectrum of compound **2**; Figure S16. ^1^H ^1^H NOESY spectrum of compound **2**.
