# Supplementary material for: Chemical Constituents from the Aerial Parts of Artemisia iwayomogi and Their Anti-Neuroinflammatory Activities
Source: Plants (Basel). 2022 Jul 27;11(15):1954. doi: 10.3390/plants11151954 (PMC9370542; doi:10.3390/plants11151954)

Supplementary data

Table of contents

Fig. S1. HR-DART-MS spectrum of compound **1**

Fig. S2. IR spectrum of compound **1**

Fig. S3. ^{1}H NMR spectrum of compound **1** (500 MHz, methanol-d_4)

Fig. S4. ^{13}C NMR spectrum of compound **1** (125 MHz, methanol-d_4)

Fig. S5. ^{1}H ^{13}C HSQC spectrum of compound **1**

Fig. S6. ^{1}H ^{1}H COSY spectrum of compound **1**

Fig. S7. ^{1}H ^{13}C HMBC spectrum of compound **1**

Fig. S8. ^{1}H ^{1}H NOESY spectrum of compound **1**

Fig. S9. HR-DART-MS spectrum of compound **2**

Fig. S10. IR spectrum of compound **2**

Fig. S11. ^{1}H NMR spectrum of compound **2** (500 MHz, methanol-d_4)

Fig. S12. ^{13}C NMR spectrum of compound **2** (125 MHz, methanol-d_4)

Fig. S13. ^{1}H ^{13}C HSQC spectrum of compound **2**

Fig. S14. ^{1}H ^{1}H COSY spectrum of compound **2**

Fig. S15. ^{1}H ^{13}C HMBC spectrum of compound **2**

Fig. S16. ^{1}H ^{1}H NOESY spectrum of compound **2**

Fig. S1. HR-DART-MS spectrum of compound **1**

Data:201027_PV1200_DV1950_300C
Sample Name:
Description:
Ionization Mode:ESI+
History:Determine m/z[Peak Detect[Centroid,25,Area];Correct Base[5.0%]];Correct Base[5.0%];Average(MS[1] 19....

Acquired:10/27/2020 12:53:36 PM
Operator:Administrator
Mass Calibration data:201027_2
Created:10/27/2020 3:51:06 PM
Created by:Administrator

Charge number:1
Tolerance:5.00(mmu)
Unsaturation Number:-1.5 .. 20.0 (Fraction:Both)
Element:^{12}C:0 .. 100, ^{1}H:0 .. 200, ^{14}N:1 .. 1, ^{16}O:0 .. 10

Mass	Intensity	Relative Intensity	Calc. Mass	Mass Difference (mmu)	Possible Formula	Unsaturation Number
338.16009	78766.00	72.30	338.16036	-0.27	$^{12}C_{17}{}^{1}H_{24}{}^{14}N_{1}{}^{16}O_{6}$	6.5

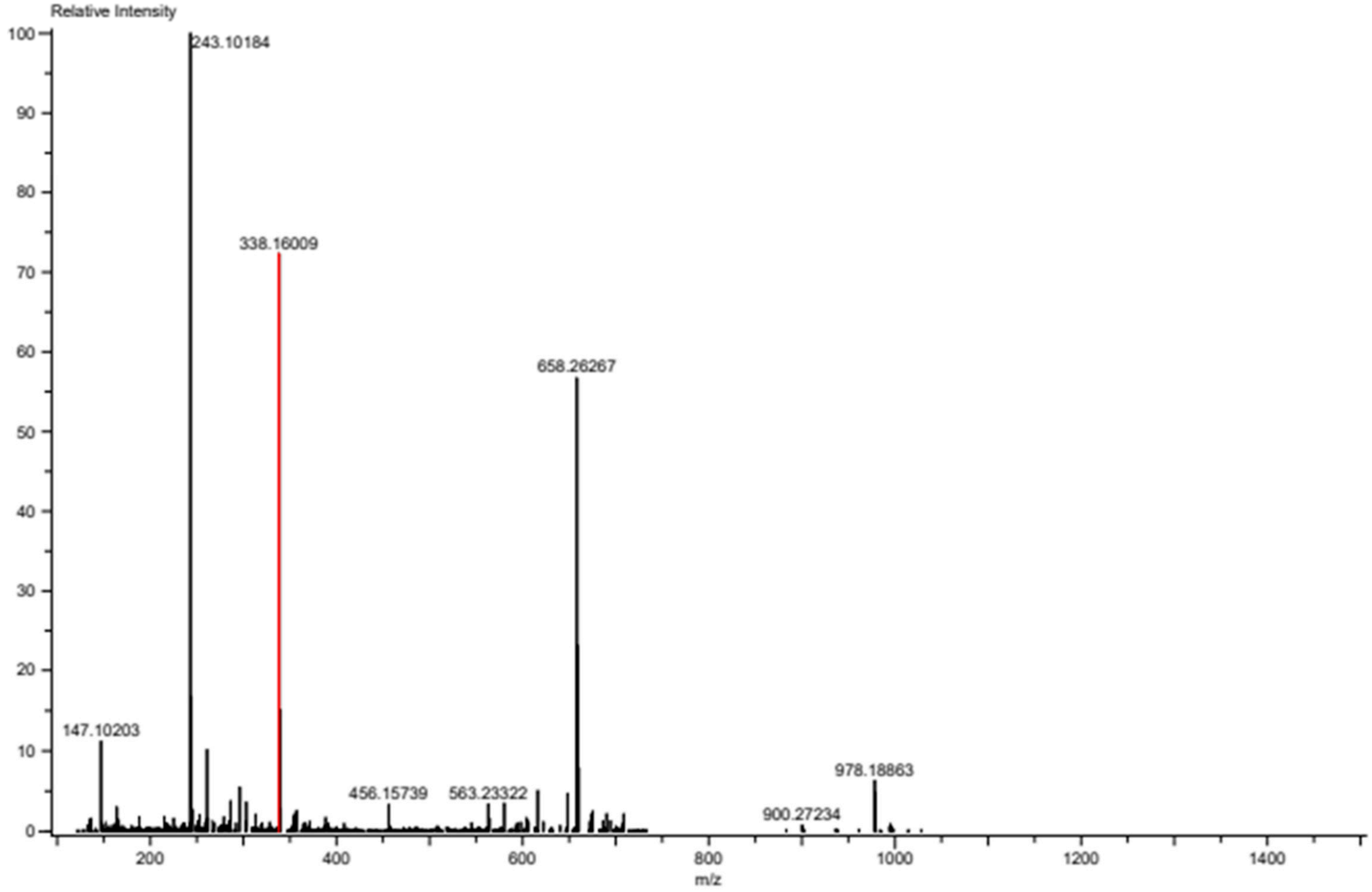

Fig. S2. IR spectrum of compound **1**

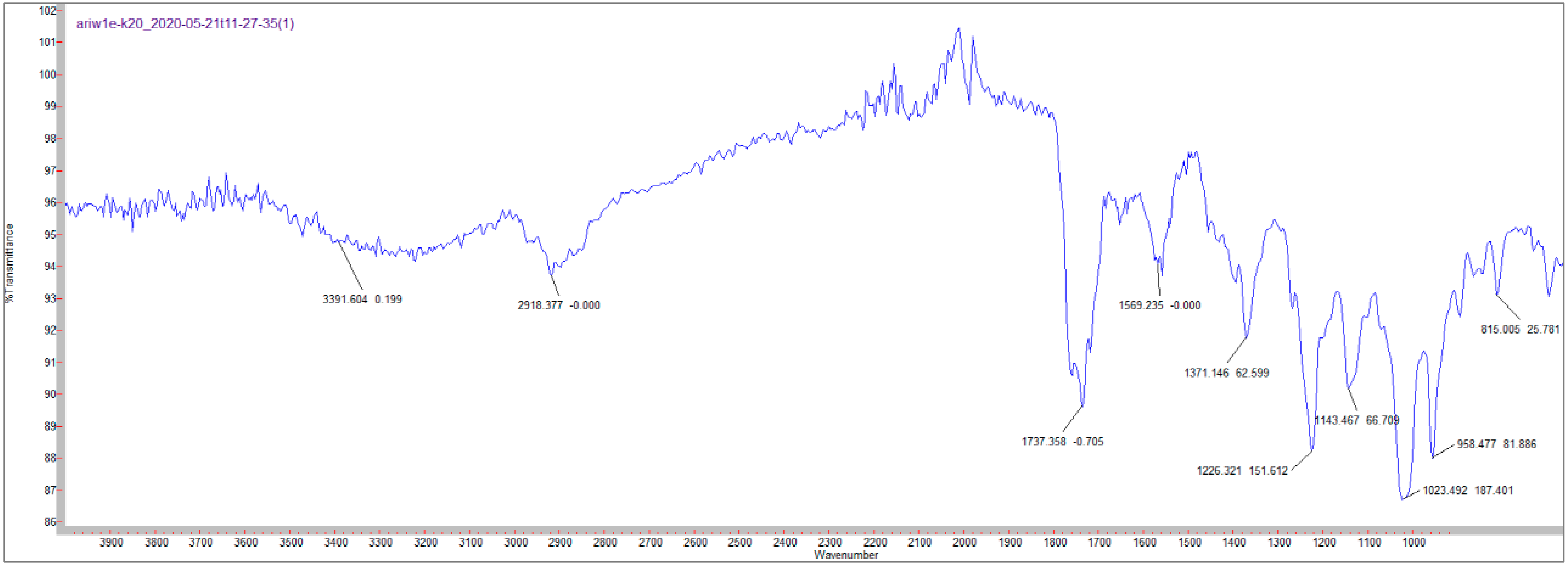

Fig. S3. ^{1}H NMR spectrum of compound **1** (500 MHz, methanol-d_4)

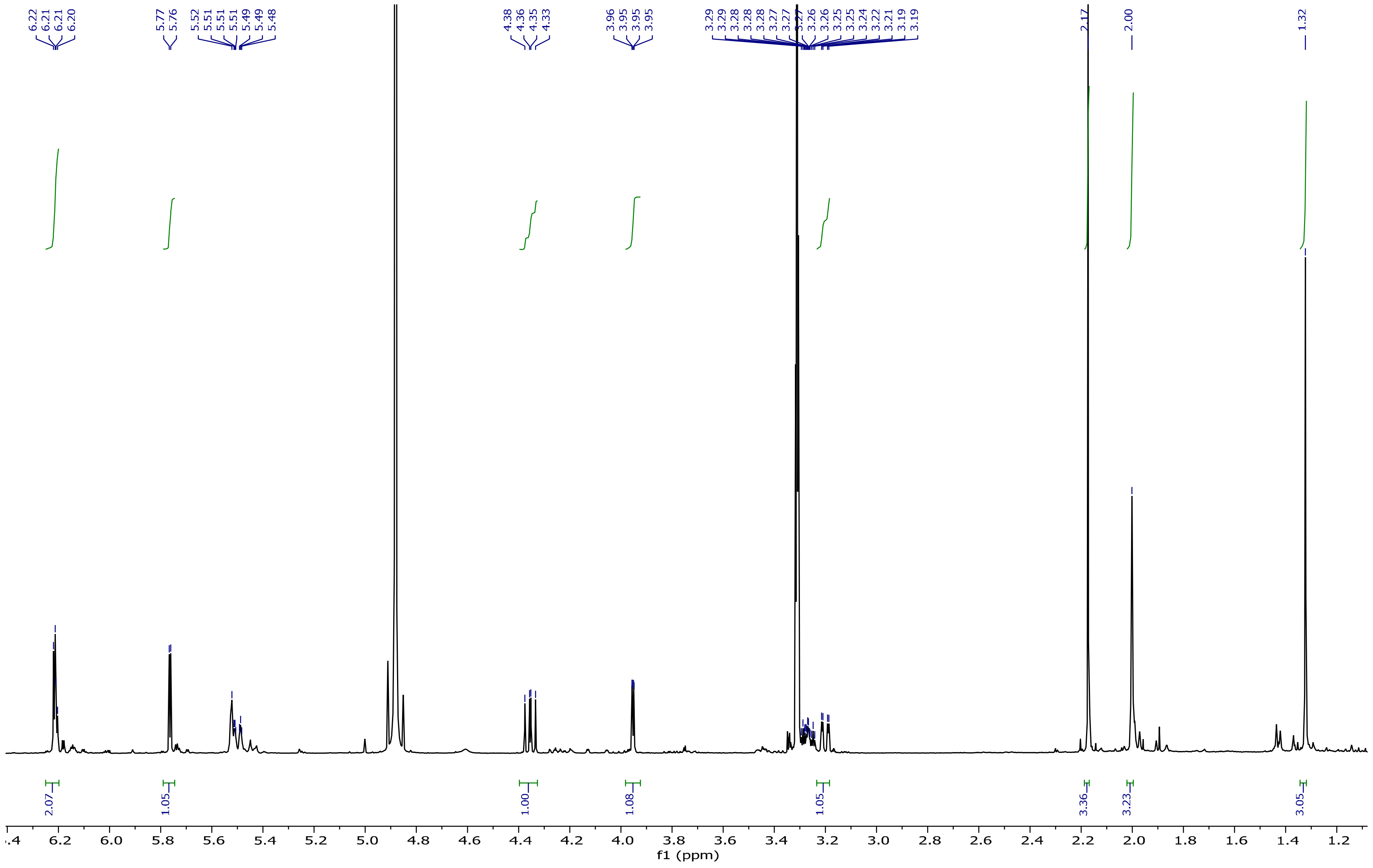

Fig. S4. ^{13}C NMR spectrum of compound **1** (125 MHz, methanol-d_4)

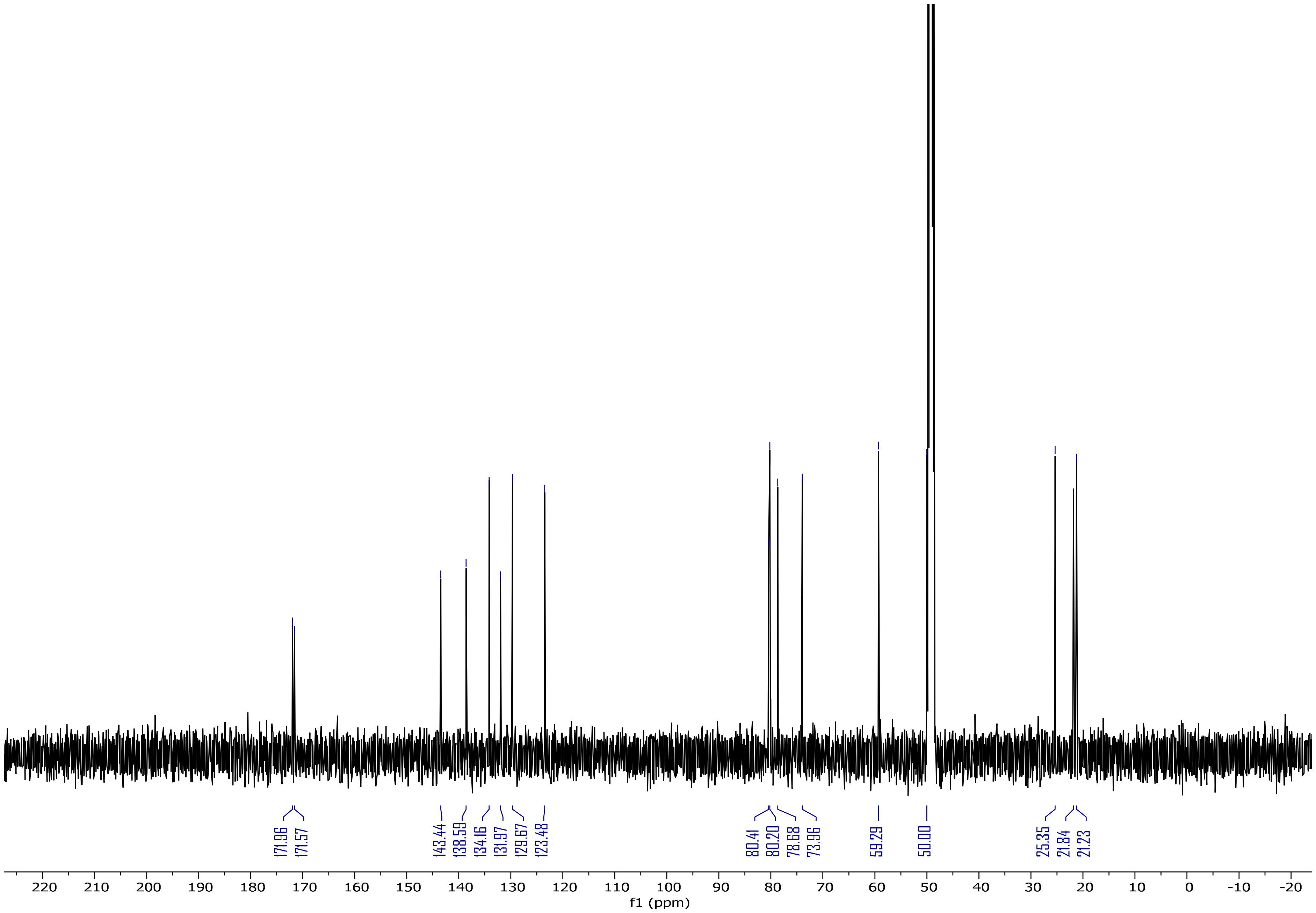

Fig. S5. ^{1}H ^{13}C HSQC spectrum of compound **1**

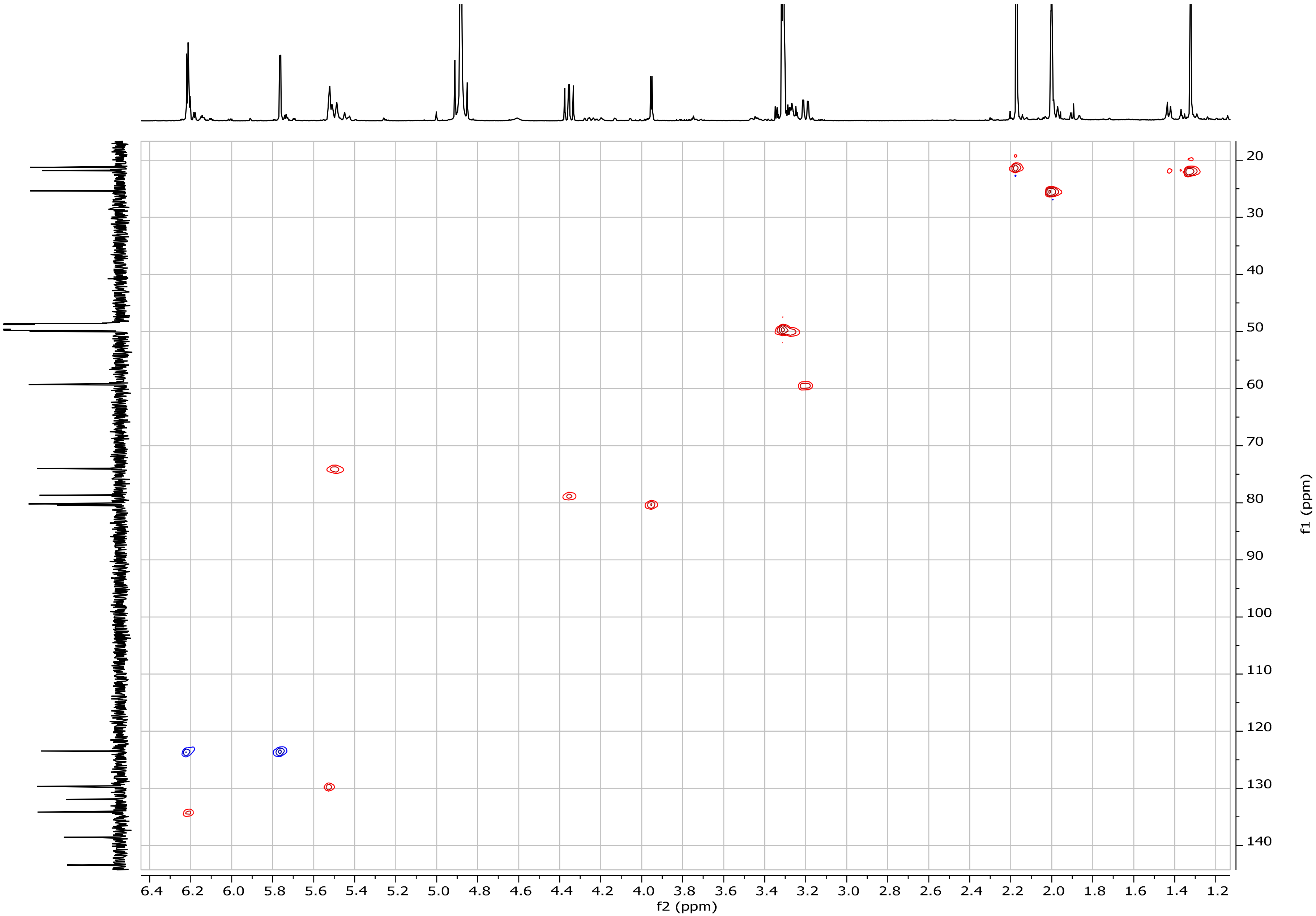

Fig. S6. ^{1}H ^{1}H COSY spectrum of compound **1**

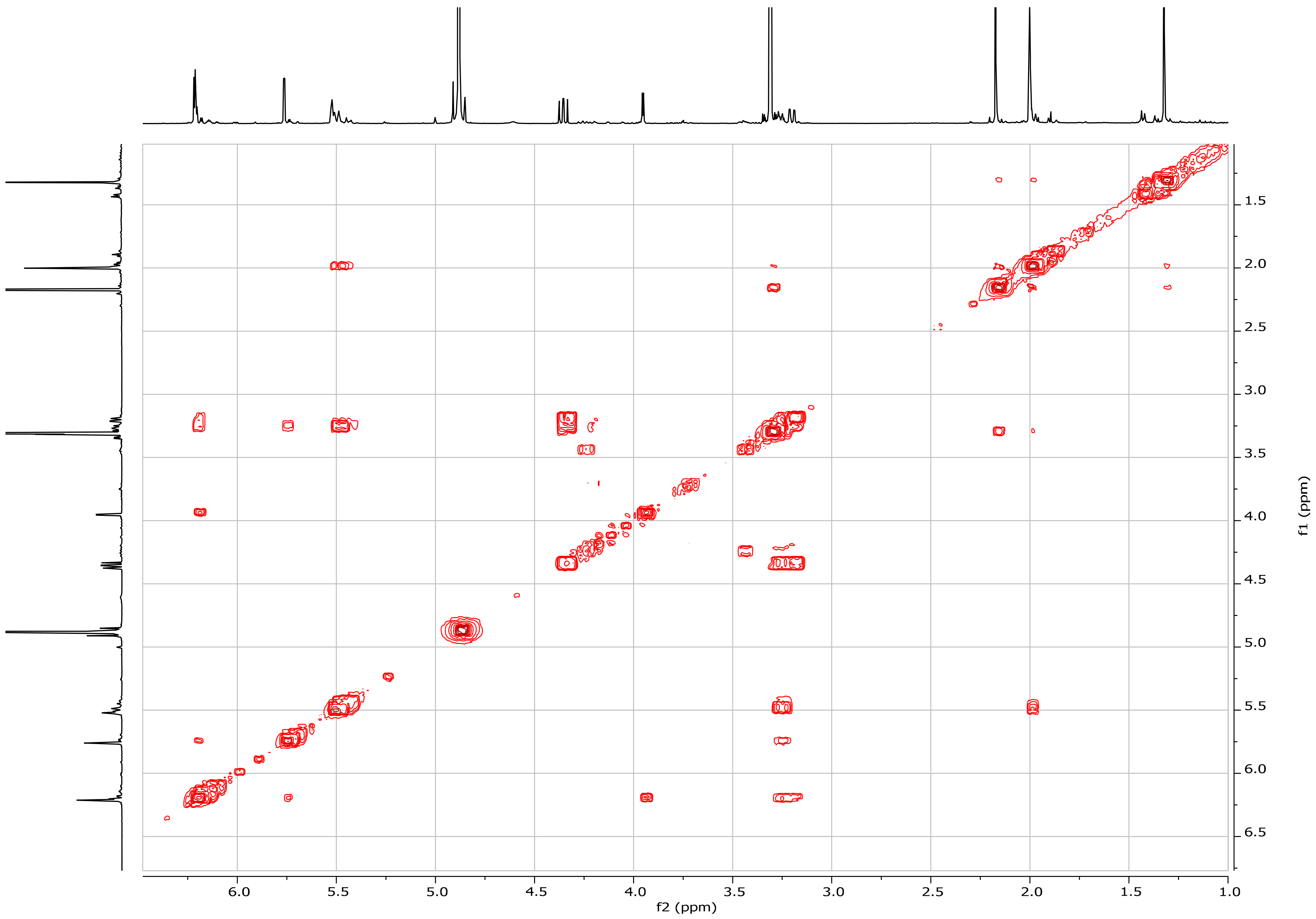

Fig. S7. ^{1}H ^{13}C HMBC spectrum of compound **1**

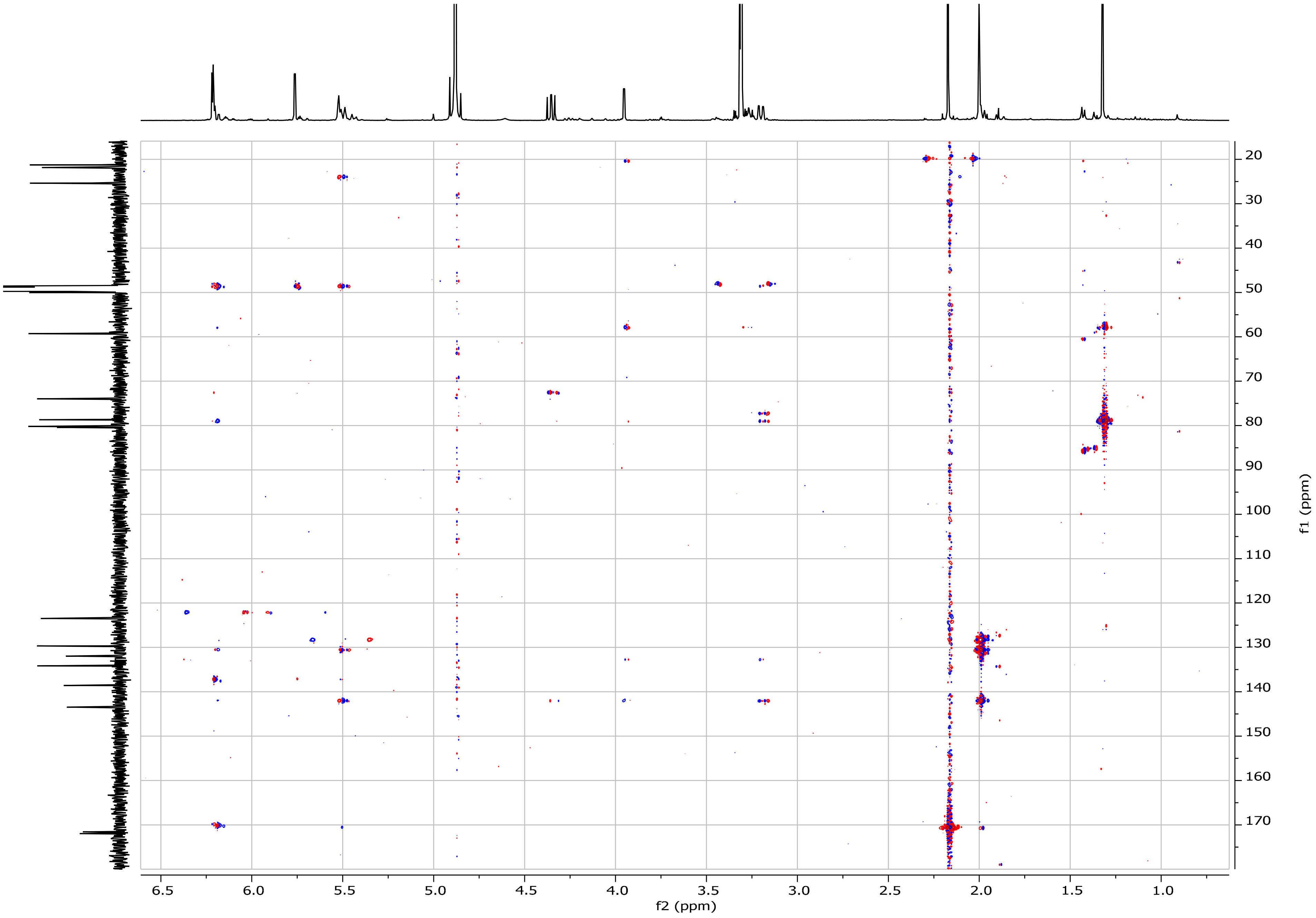

Fig. S8. ^{1}H ^{1}H NOESY spectrum of compound **1**

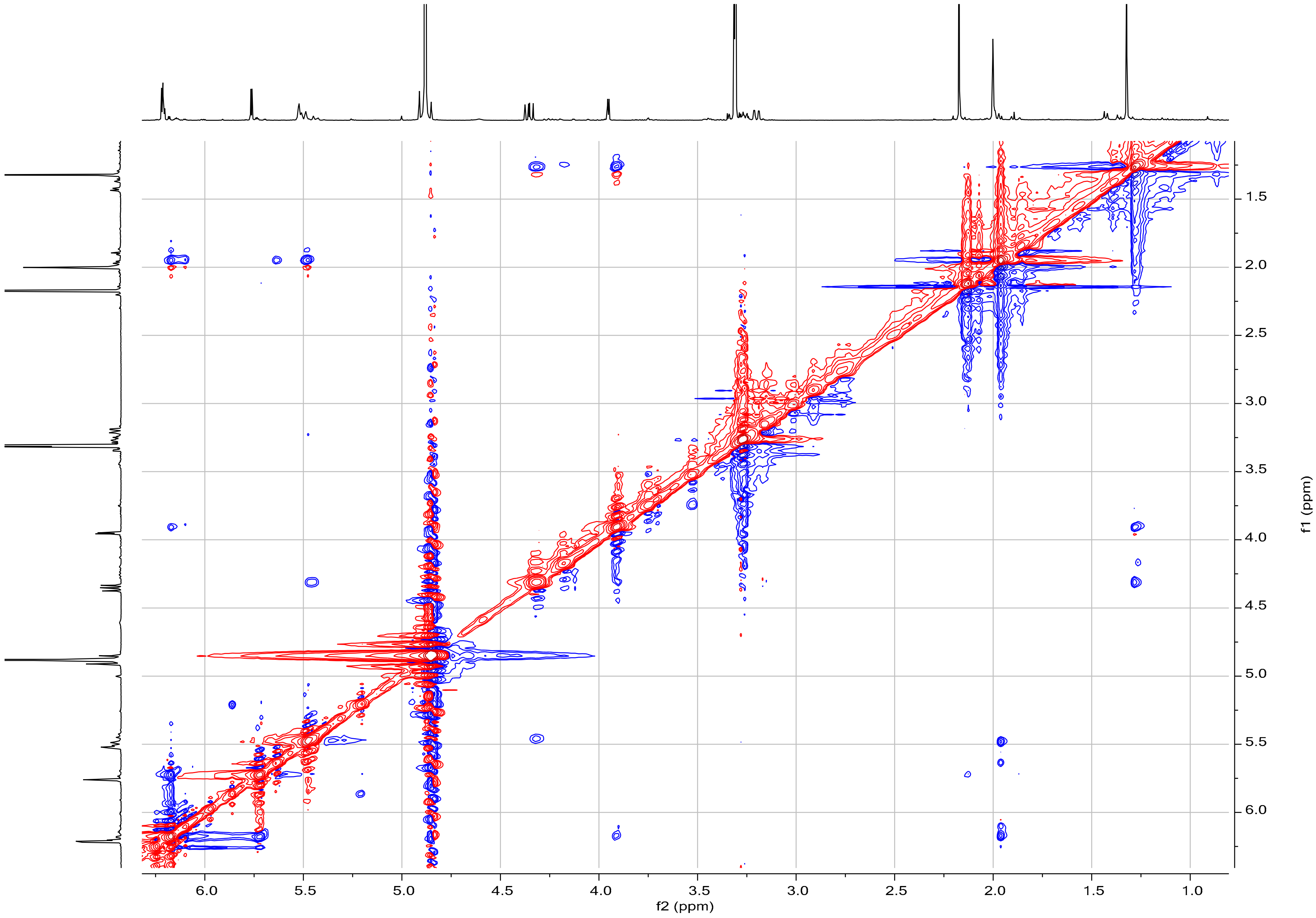

Fig. S9. HR-DART-MS spectrum of compound **2**

Data:201027_PV1200_DV1950_300C
Sample Name:
Description:
Ionization Mode:ESI+
History:Determine m/z[Peak Detect[Centroid,25,Area];Correct Base[5.0%]];Correct Base[5.0%];Average(MS[1] 18....

Acquired:10/27/2020 12:53:36 PM
Operator:Administrator
Mass Calibration data:201027_2
Created:10/27/2020 3:48:40 PM
Created by:Administrator

Charge number:1
Tolerance:5.00(mmu)
Unsaturation Number:-1.5 .. 20.0 (Fraction:Both)
Element:^{12}C:0 .. 100, ^{1}H:0 .. 200, ^{14}N:1 .. 1, ^{16}O:0 .. 10

Mass	Intensity	Relative Intensity	Calc. Mass	Mass Difference (mmu)	Possible Formula	Unsaturation Number
338.16007	62399.44	100.00	338.16036	-0.29	$^{12}C_{17}{}^{1}H_{24}{}^{14}N_{1}{}^{16}O_{6}$	6.5

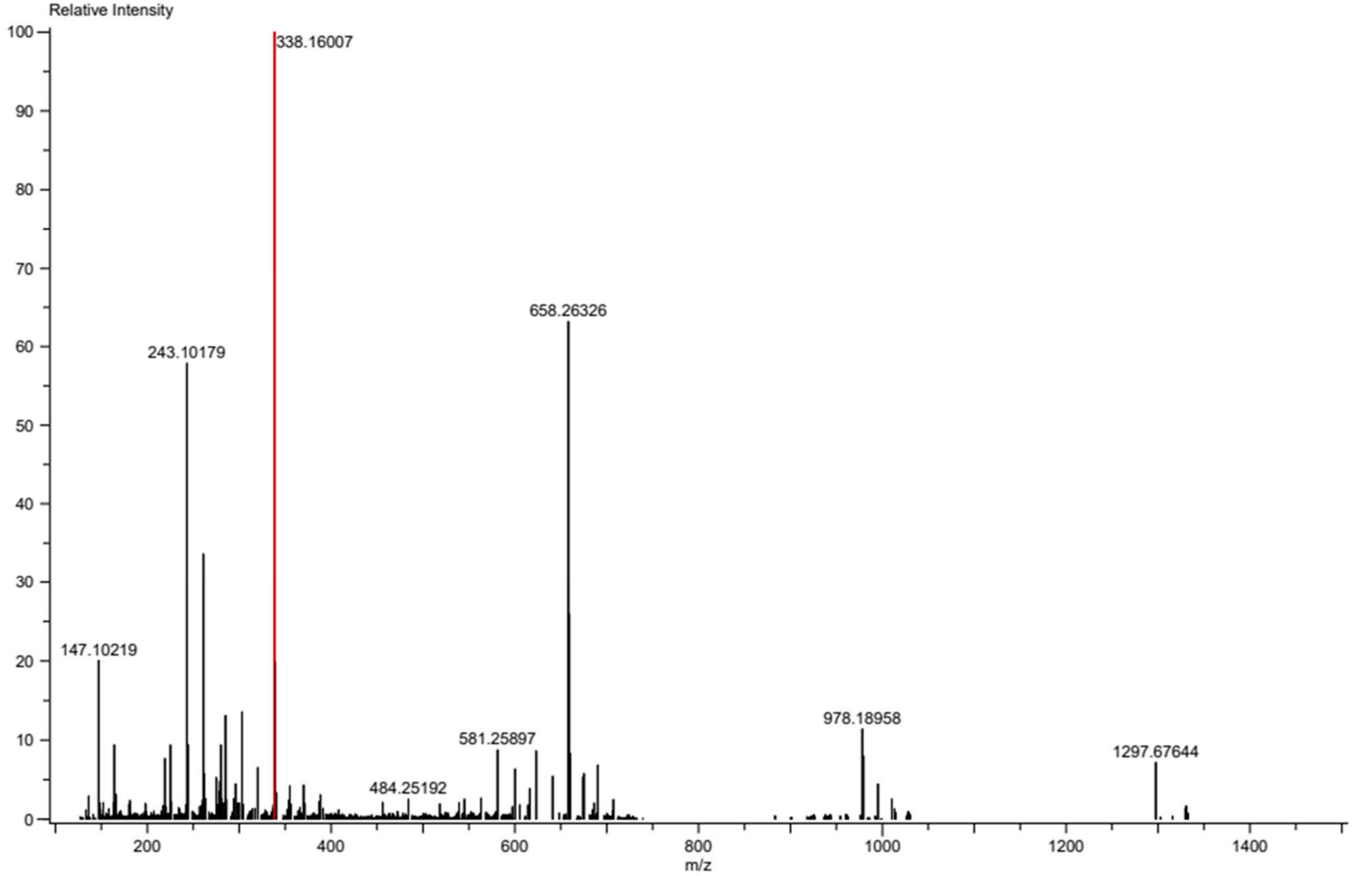

Fig. S10. IR spectrum of compound **2**

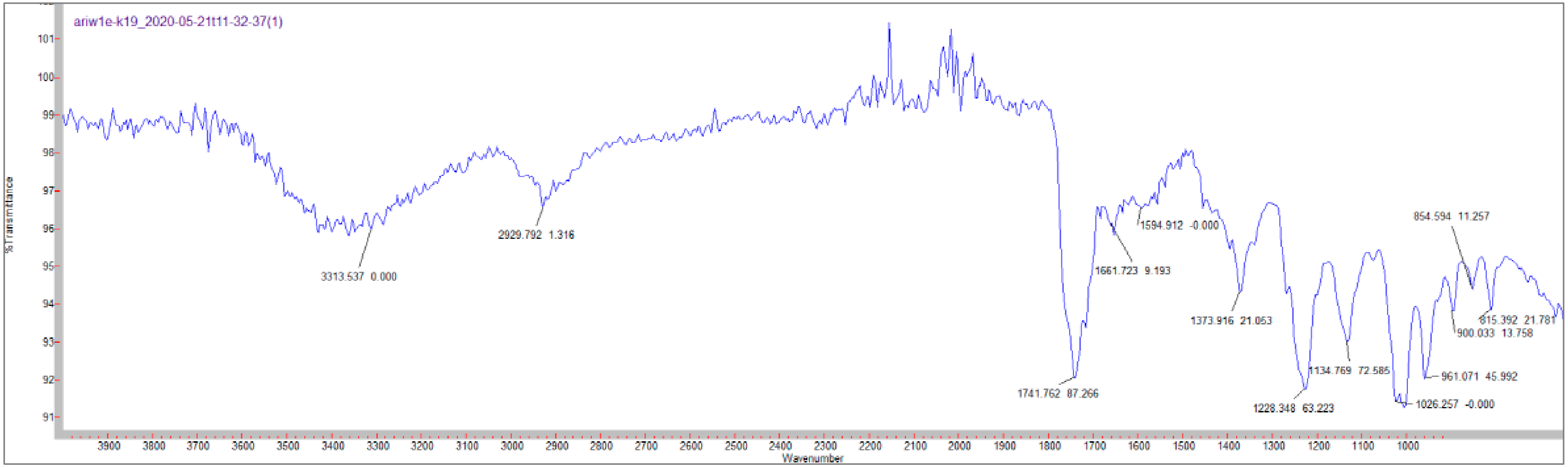

Fig. S11. ^{1}H NMR spectrum of compound **2** (500 MHz, methanol-d_4)

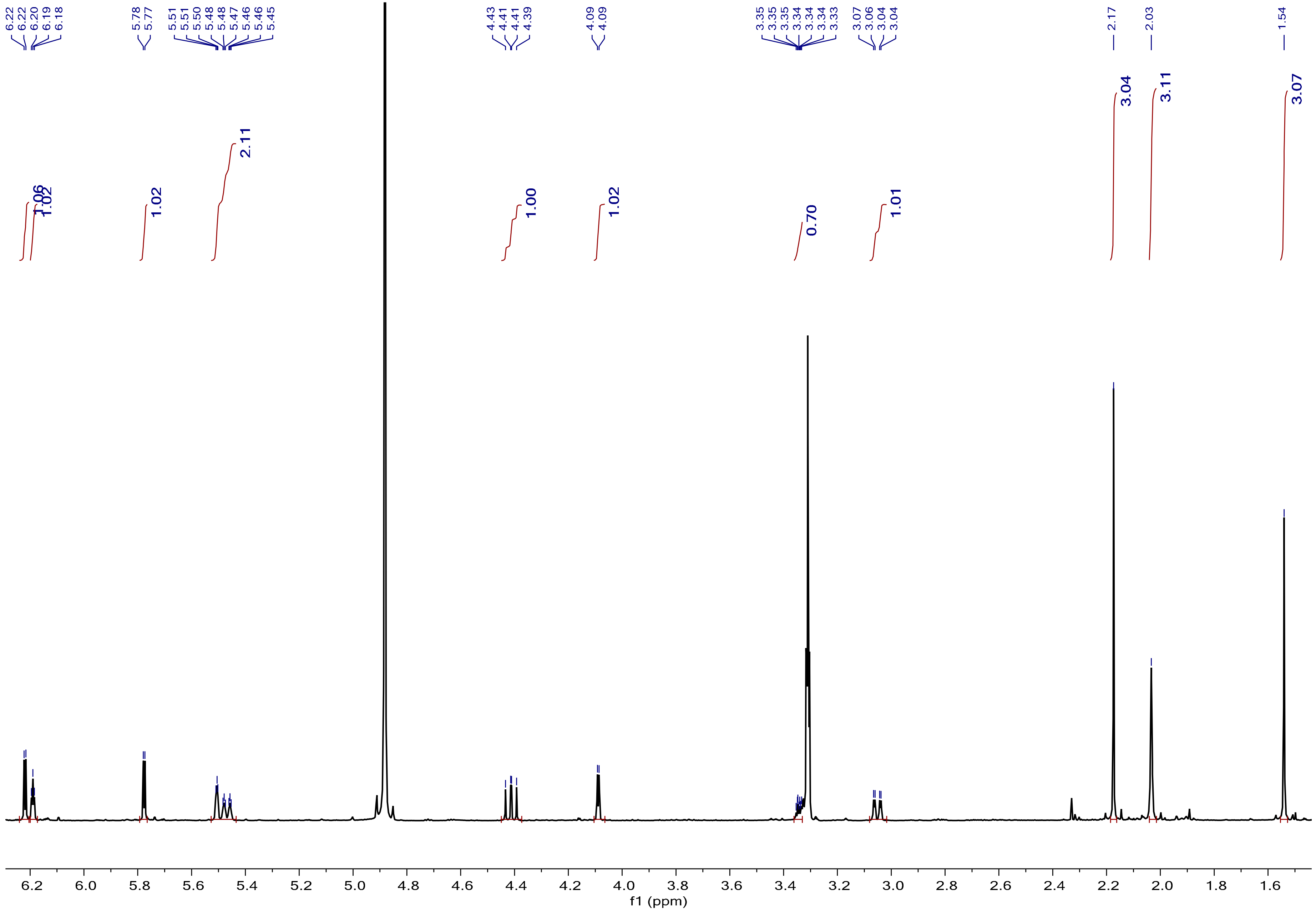

Fig. S12. ^{13}C NMR spectrum of compound **2** (125 MHz, methanol-d_4)

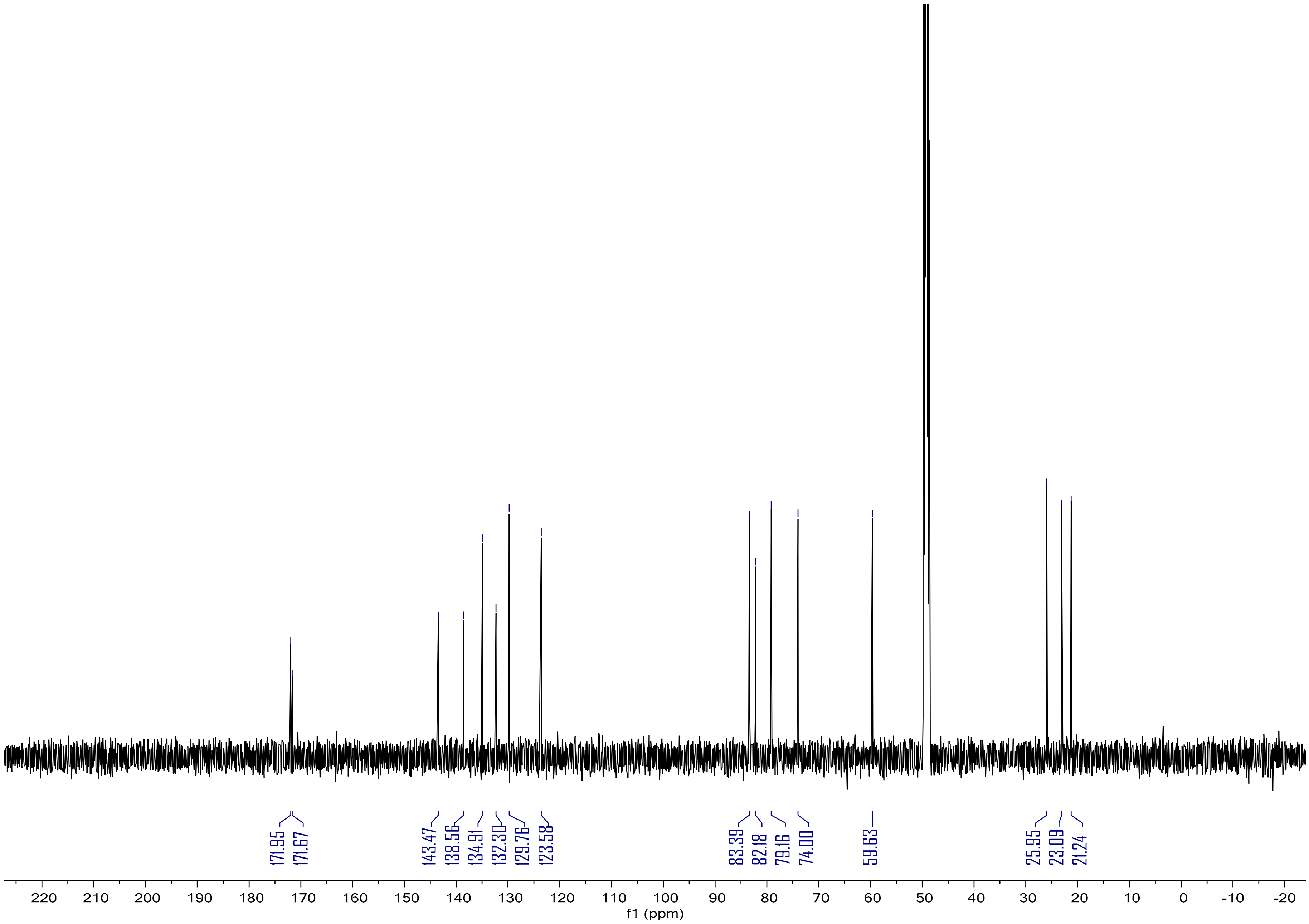

Fig. S13. ^{1}H ^{13}C HSQC spectrum of compound **2**

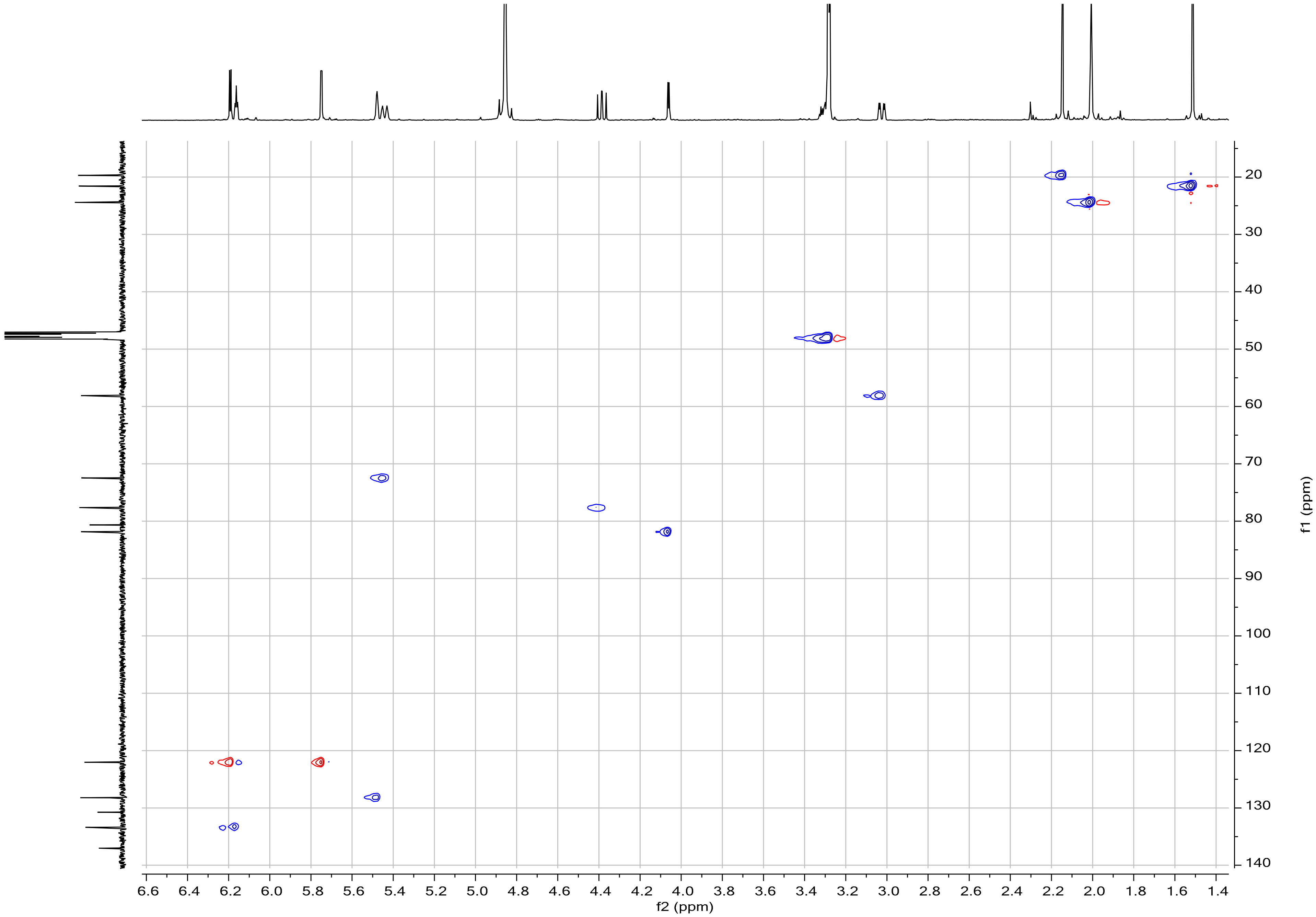

Fig. S14. ^{1}H ^{1}H COSY spectrum of compound **2**

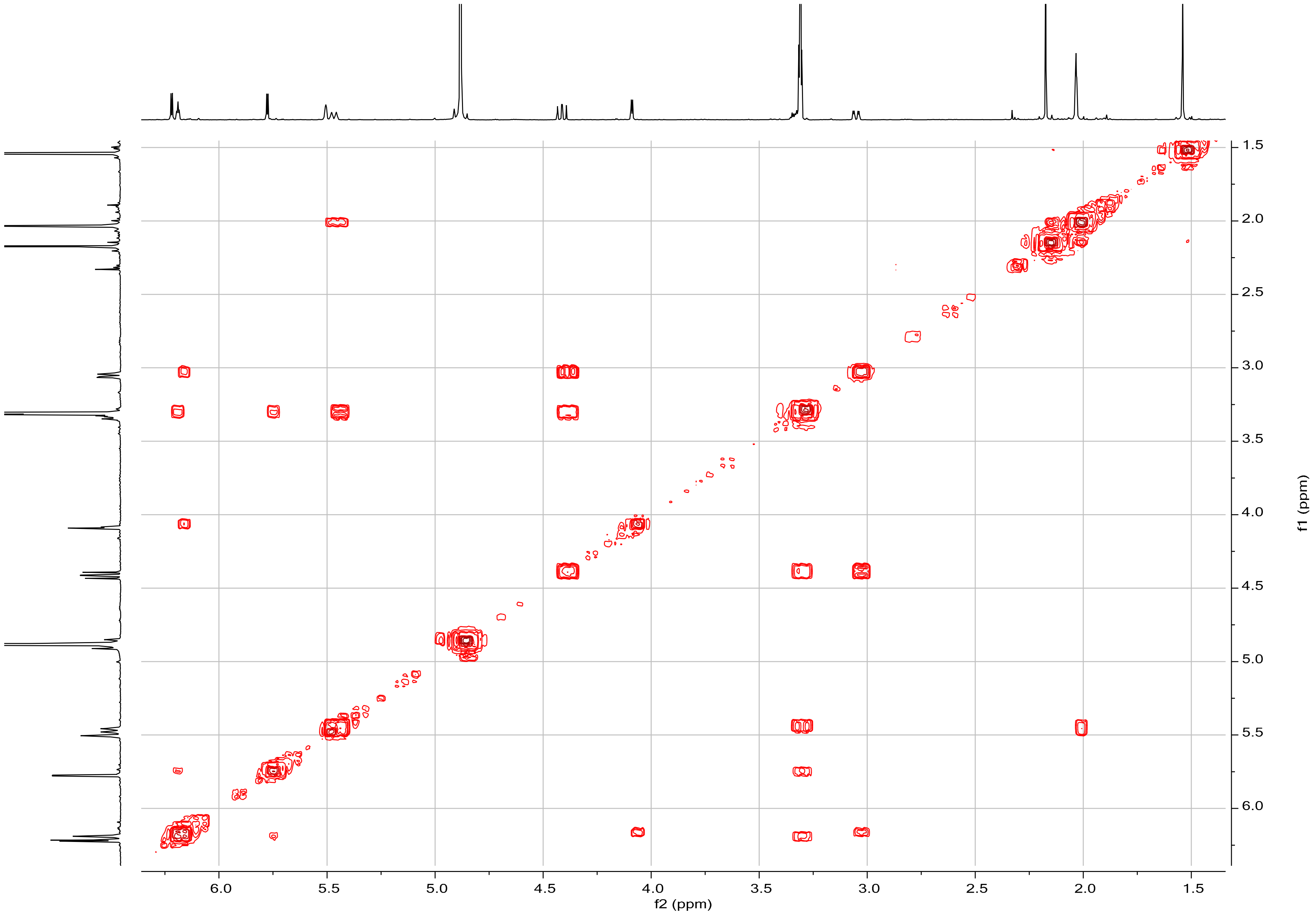

Fig. S15. ^{1}H ^{13}C HMBC spectrum of compound **2**

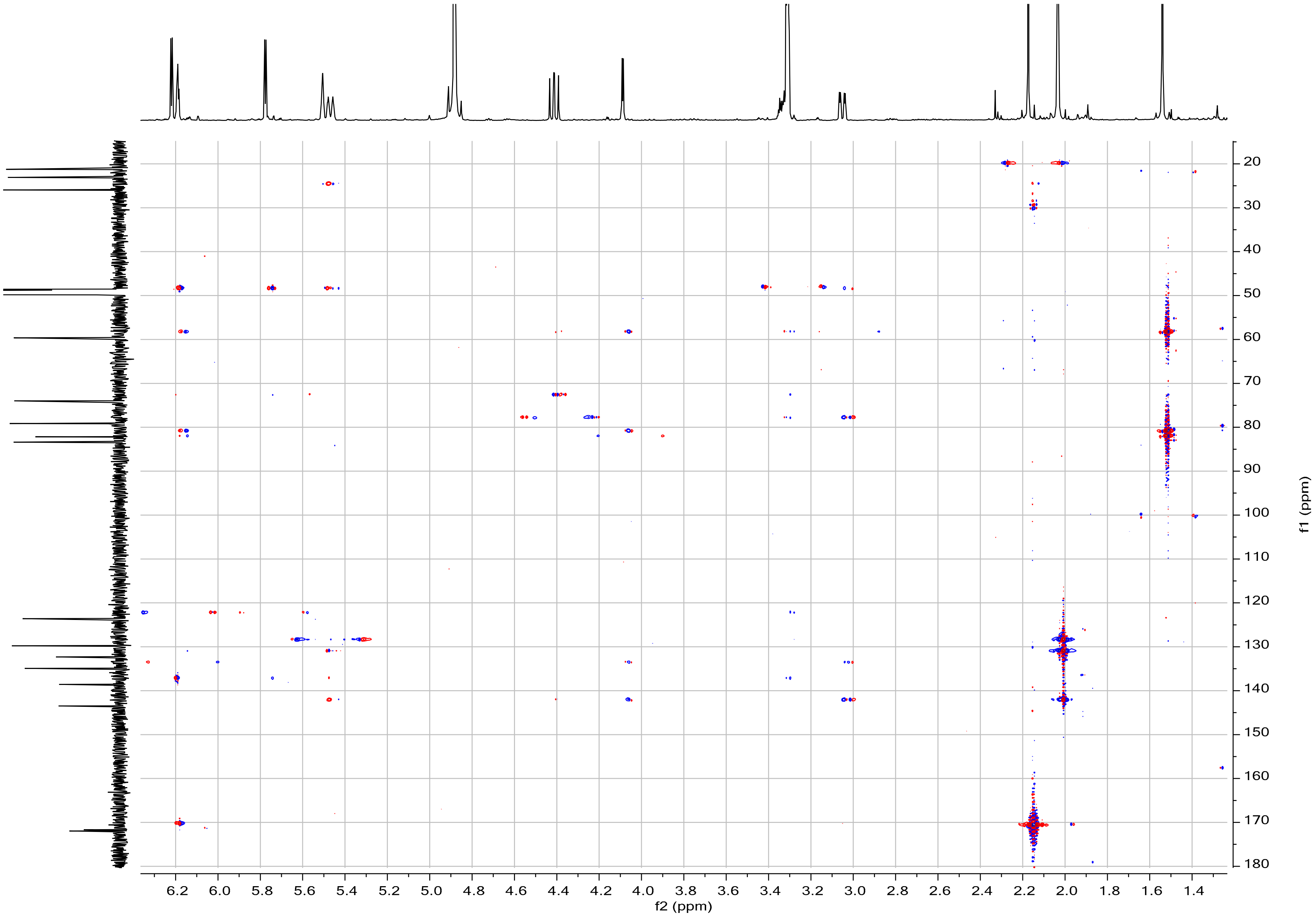

Fig. S16. ^1H ^1H NOESY spectrum of compound **2**

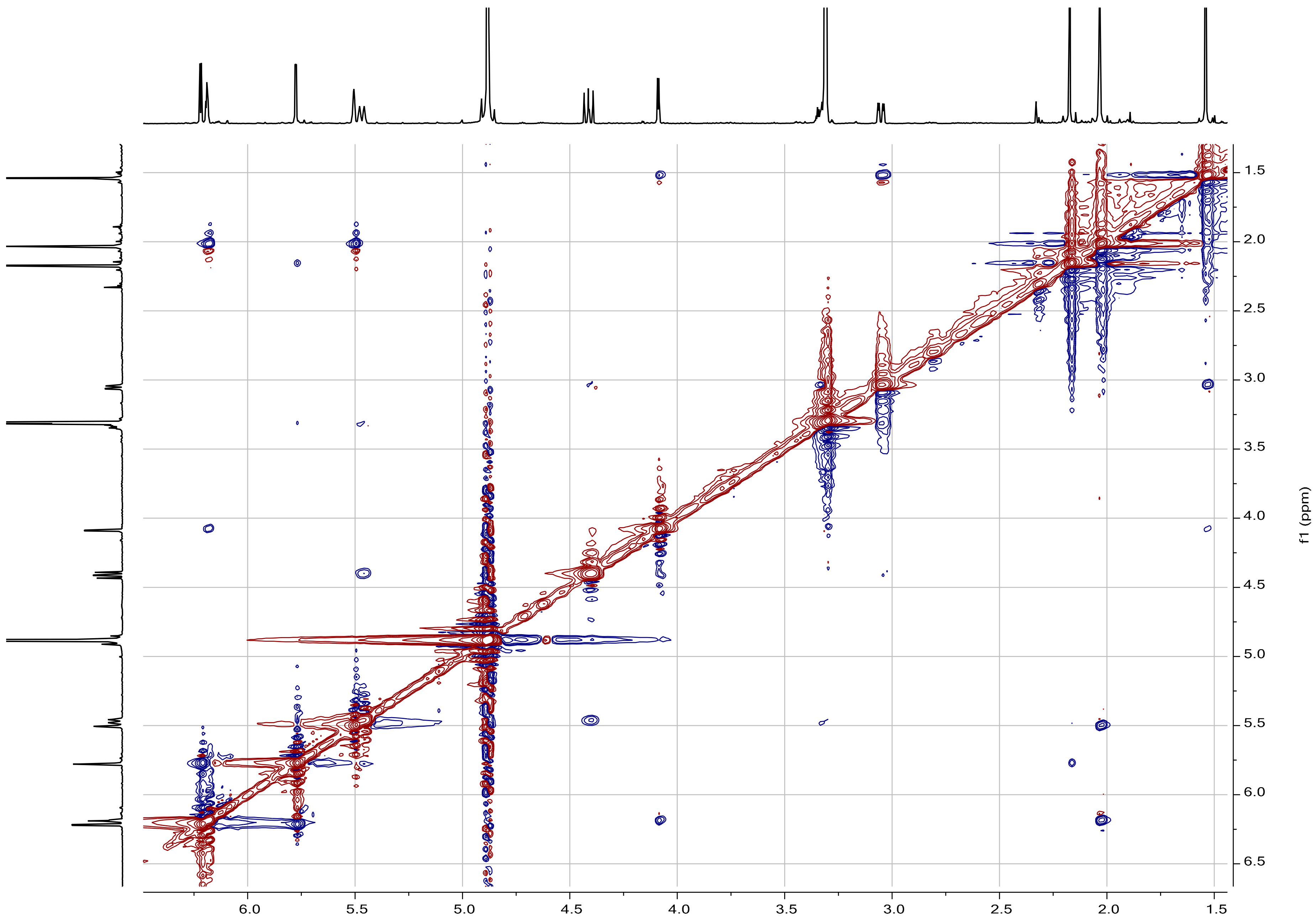

Supplement: Supplementary file 1 [file plants-11-01954-s001.zip › plants-1836489-supplementary.pdf]
